# Expression of Antizyme Inhibitor 2 in Mast Cells and Role of Polyamines as Selective Regulators of Serotonin Secretion

**DOI:** 10.1371/journal.pone.0006858

**Published:** 2009-08-31

**Authors:** Kristiina Kanerva, Jani Lappalainen, Laura T. Mäkitie, Susanna Virolainen, Petri T. Kovanen, Leif C. Andersson

**Affiliations:** 1 Department of Pathology, Haartman Institute, University of Helsinki, Helsinki, Finland; 2 Wihuri Research Institute, Helsinki, Finland; 3 HUSLAB, Helsinki, Finland; Cairo University, Egypt

## Abstract

**Background:**

Upon IgE-mediated activation, mast cells (MC) exocytose their cytoplasmic secretory granules and release a variety of bioactive substances that trigger inflammatory responses. Polyamines mediate numerous cellular and physiological functions. We report here that MCs express antizyme inhibitor 2 (AZIN2), an activator of polyamine biosynthesis, previously reported to be exclusively expressed in the brain and testis. We have investigated the intracellular localization of AZIN2 both in resting and activated MCs. In addition, we have examined the functional role of polyamines, downstream effectors of AZIN2, as potential regulators of MC activity.

**Methodology/Principal Findings:**

Immunostainings show that AZIN2 is expressed in primary and neoplastic human and rodent MCs. We demonstrate that AZIN2 localizes in the Vamp-8 positive, serotonin-containing subset of MC granules, but not in tryptase-containing granules, as revealed by double immunofluorescence stainings. Furthermore, activation of MCs induces rapid upregulation of AZIN2 expression and its redistribution, suggesting a role for AZIN2 in secretory granule exocytosis. We also demonstrate that release of serotonin from activated MCs is polyamine-dependent whereas release of histamine and β-hexosaminidase is not, indicating a granule subtype-specific function for polyamines.

**Conclusions/Significance:**

The study reports for the first time the expression of AZIN2 outside the brain and testis, and demonstrates the intracellular localization of endogenous AZIN2 in MCs. The granule subtype-specific expression and its induction after MC activation suggest a role for AZIN2 as a local, *in situ* regulator of polyamine biosynthesis in association with serotonin-containing granules of MCs. Furthermore, our data indicates a novel function for polyamines as selective regulators of serotonin release from MCs.

## Introduction

Mast cells (MC) are highly versatile effector cells of the adaptive and the innate immune system [1], [2]. They are involved in a variety of reactions ranging from acute allergic responses to chronic inflammations, autoimmune disorders, atherosclerosis, and cancer. Mammalian MCs are widely distributed in virtually all vascularized tissues, and they are especially numerous in anatomical sites directly interfacing with the external environment, such as the skin, the airways, and the gastrointestinal tract. MCs originate from bone marrow hematopoietic CD34+ progenitor cells, which enter the circulation and migrate to peripheral tissues where they ultimately reside. In tissues, MCs undergo differentiation in the local cytokine microenvironment [3]. Mature MCs thus represent a heterogenous population of cells with various phenotypes depending on the anatomical location [4]. On their surface, MCs express FcεRI, a high-affinity IgE receptor, which upon activation induces exocytosis of cytoplasmic secretory granules, *de novo* synthesis of lipid-derived mediators, and release of cytokines, chemokines, and growth factors [5]. Their membrane-bound secretory granules contain a variety of preformed mediators such as histamine, serotonin, and proteases such as tryptase and chymase [6]. MCs are involved in various pathological conditions including mastocytosis, which is a spectrum of heterogenous and rare disorders characterized by the pathological expansion and accumulation of MCs in tissues [6]. The most prevalent form of mastocytosis is cutaneous mastocytosis, in which MC infiltrations are found in skin.

Polyamines play a pivotal role in cell proliferation, apoptosis and ion-channel gating. The cellular pools of polyamines are under strict control; their uptake, biosynthesis, and catabolism are regulated carefully [7]. In mammalian cells, ornithine decarboxylase (ODC) catalyzes the rate-limiting step in polyamine biosynthesis. Polyamine-induced antizymes (AZ) are the key proteins that regulate the uptake of polyamines and the activity of ODC [8]. Active ODC homodimers are converted to inactive AZ-bound ODC-monomers which are concomitantly degraded by 26S proteasome in a ubiquitin-independent manner [9]. So far, three paralogs of mammalian AZ molecules have been described [10], [11]. AZ itself is under a multilevel control, achieved at protein level by an ODC-homologous protein, namely antizyme inhibitor (AZIN). AZIN binds AZ with higher affinity than ODC hence inducing activation of ODC [12], [13]. Two discrete AZINs are expressed in mammalian cells, AZIN1 and AZIN2, both of which counteract the functions of AZ thereby activating ODC and depressing its degradation as well as enhancing polyamine uptake [14]–[18]. All mammalian cells express the initially characterized AZIN1, whereas AZIN2 expression has been reported to be most abundant in brain and testis [19]. AZIN2 has been shown to have lower affinity than AZIN1 towards AZs [18] but it is not known why both AZINs are present in certain types of cells.

Upregulation of ODC activity and excess of cellular polyamines are associated with rapidly dividing cells and neoplastic growth [20]. However, much less attention has been paid to polyamine functions in terminally differentiated, non-proliferating cells such as MCs. It has nevertheless been shown that polyamines stimulate exocytosis of MC granules by activating small GTP-binding proteins [21], but the molecular mechanisms contributing to granule exocytosis have remained unknown and the role of polyamines in these cells is unsettled.

In the present study, we raised two rabbit antisera against AZIN2 and immunostained sections of various human tissues with them. We found that human skin MCs stained strongly positive for AZIN2. We therefore decided to investigate the functional role of AZIN2 and polyamines as potential mediators of MC activity. Our data show that AZIN2 specifically localizes in the Vamp-8 positive, serotonin-containing granules and that activation of ODC is selectively associated with the secretion of serotonin, but not with the secretion of β-hexosaminidase (β-hex) or histamine from human MCs. Our findings thus suggest that AZIN2 might function as *in situ* regulator of ODC in association with MC granules.

## Materials and Methods

### Chemicals and supplies

All of the chemicals used were obtained from Sigma (St. Louis, MO, USA) unless stated otherwise. Oligonucleotides were purchased from Oligomer (Helsinki, Finland), Lipofectamine 2000 was from Invitrogen (Carlsbad, CA, USA), the Complete protease inhibitor cocktail was from Roche (Mannheim, Germany), and D,L-α-difluoromethylornithine (DFMO) was from Merrell Dow Research Institute (Cincinnati, OH, USA). The following antibodies were used: mouse anti-FLAG M2 (Sigma-Aldrich); mouse anti-human tryptase (Dako Cytomation, Glostrup, Denmark); mouse anti-human Vamp-8 (Abnova, Taipei City, Taiwan); mouse anti-human serotonin (Dako Cytomation); goat anti-rabbit Alexa Fluor-680, donkey anti-rabbit Alexa Fluor-555, and donkey anti-mouse Alexa Fluor-488 (all from Invitrogen Molecular Probes, Eugen, OR, USA); donkey anti-mouse IRDye 800CW (LI-COR Biosciences, Lincoln, NE, USA).

### Production and characterization of AZIN2 antibodies

Two polyclonal rabbit antisera were raised against synthetic peptides STRDLLKELTLGASQATTDEVA (antiserum 2) and STRDLLKELTLGASQATT (antiserum 3), corresponding to amino acids 18–39 and 18–35 of AZIN2, respectively (Genbank accession No. NP_443724). This N-terminal sequence has low homology to ODC and AZIN1. Branched peptides were synthesized with an automated multiple peptide synthesizer (MultiPep; Intavis Bioanalytical Instruments AG, Cologne, Germany) using Fmoc-chemistry and purified with HPLC using a Supelco Discovery Biowide Pore C18 column (Supelco, Bellefonte, PA, USA). The main peaks were collected, lyophilized and used as antigens for immunizations. The antibodies were produced at at the Viikki Laboratory Animal Centre, University of Helsinki, Finland. All animals were handled in strict accordance with good animal practice as defined by the relevant Finnish animal welfare bodies, and the European Communities Council directive (86/609/EEC). All animal work was approved by the Animal Experiment Board of the State Provincial Office of Southern Finland (Reference number HY176-02) in accordance with national legislation. Whole sera from the immunized rabbits were used for immunofluorescence stainings and negative controls were prepared by substituting the primary antibodies with preimmune rabbit sera.

The cDNAs encoding AZIN2, AZIN1, and ODC were amplified from the corresponding constructs described earlier [16] and they were subcloned into the p3xFLAG-CMV-10 vector (Sigma-Aldrich). COS-7 cells were grown to 50% confluence and transfected for 24 h with plasmids carrying FLAG-tagged AZIN2, AZIN1, or ODC gene or an empty FLAG-vector using Lipofectamine. The cells were lysed in buffer (50 mM Hepes, pH 7.0, 150 mM NaCl, 10% glycerol, 1% Triton X-100, 1.5 mM EGTA, 100 mM NaF, 10 mM Na_4_P_2_O_7_ with protease inhibitors) and the proteins were resolved by 12% SDS-PAGE and transferred to nitrocellulose membrane (Bio-Rad, Hercules, CA, USA). The membranes were blocked with Odyssey blocking buffer (LI-COR Biosciences), incubated with rabbit AZIN2 antiserum 2 or 3 (diluted 1∶200) together with mouse anti-FLAG M2 antibody (3 µg/ml), followed by donkey anti-mouse IRDye 800CW and goat anti-rabbit Alexa Fluor-680 secondary antibodies (both diluted 1∶10,000). An Odyssey Infrared Imager (LI-COR Biosciences) was used for visualization.

### Tissue samples

Formalin-fixed, paraffin-embedded tissue samples of human skin from patients diagnosed with cutaneous mastocytosis were obtained from the Skin and Allergy Hospital, Helsinki University Central Hospital, Helsinki, Finland.

#### Ethics Statement

This study was conducted according to the principles expressed in the Declaration of Helsinki. All the tissue samples were obtained with written informed consent from the patients or their guardians. The tissue samples were used for this study under approval of the institutional review board of Haartman Institute, University of Helsinki.

### Cell culture and activation

Rat basophilic leukemia cell line RBL-1 [22] was provided by Dr. Tomas Mustelin (The Burnham Institute for Medical Research, La Jolla, CA, USA) and the cells were cultured in RPMI 1640 (Gibco BRL, Carlsbad, CA, USA) medium supplemented with 10% (v/v) fetal calf serum (Gibco), 1 mM L-glutamine (Fluka, Buchs, Switzerland), and antibiotics. All cell cultures were maintained at 37°C in a humidified 5% CO_2_ atmosphere. The immature human leukemic MC line HMC-1 was provided by Dr. Joseph H. Butterfield (Mayo Clinic, Rochester, NY, USA) and maintained as previously described [23]. The human MC line LAD2, originating from a patient with untreated MC sarcoma, was provided by Dr. Dean Metcalfe (National Institute of Allergy and Infectious Disease, National Institutes of Health, Bethesda, MD, USA) and cultured as previously described [24]. Peripheral blood-derived, human MCs were cultured *in vitro* as described earlier [25].

The MCs were activated by 50 nM phorbol 12-myristate 13-acetate (PMA) together with 1 µM (HMC-1) or 2 µM (RBL-1) calcium ionophore A23187, or with 1 µg/ml of goat anti-human IgE (primary human MCs). The cells were fixed or collected at the indicated times and processed for immunocytochemical stainings or quantitative real-time RT-PCR.

### Immunohisto- and immunocytochemistry

Immunohistochemistry was carried out on 4–5 µm-thick skin tissue sections from paraffin-embedded samples obtained from patients with cutaneous mastocytosis (n = 15). The sections were air-dried, deparaffinized in xylene, and rehydrated in a graded ethanol series. Antigens were retrieved by microwaving sections in 10 mM citrate buffer (pH 6) for 20 min. Endogenous peroxidase activity was quenched with 0.5% hydrogen peroxide in methanol for 30 min, after which the sections were incubated with CAS-block (Zymed Laboratories, Carlsbad, CA, USA) to block non-specific reactivity. The AZIN2 antiserum 2 was diluted 1∶180–1∶300, and AZIN2 antiserum 3 diluted 1∶500–1∶600 in DakoREAL antibody diluent (Dako, Glostrup, Denmark) and incubated overnight at +4°C. Bound antibodies were detected with an avidin-biotin-horseradish peroxidase complex using a Vectastain Elite ABC kit (Vector Laboratories, Burlingame, CA, USA) and visualized by 3-amino-9-ethyl carbazole. The slides were finally counterstained with hematoxylin and mounted. Negative controls were prepared by substituting the primary antibody with the corresponding preimmune rabbit serum.

For immunocytochemical staining, the MCs were cytocentrifuged with a Cytospin 3 centrifuge (ThermoShandon, Pittsburgh, PA, USA) and fixed with MeOH. The staining was otherwise carried out as specified above, except that AZIN2 antiserum 2 was diluted 1∶100 and AZIN2 antiserum 3 was diluted 1∶300. Light microscopy was performed with an Olympus BX51 microscope (Olympus Optical, Tokyo, Japan) and images were acquired with a Nikon Digital Sight DS-5M camera (Nikon Corporation, Tokyo, Japan) using NIS-Elements F 2.30 software (Nikon Corporation). The cells were photographed with a 40× objective (numeric aperture 0.95) or a 100× oil objective (numeric aperture 1.25).

For immunofluorescence staining, cytocentrifuged LAD2 and RBL-1 cells were fixed with ice-cold methanol for 10 min, and blocked with 10% human serum in PBS for 30 min. They were then incubated with primary antibodies and species-appropriate secondary antibodies conjugated with either Alexa Fluor-488 or Alexa Fluor-555. Samples were mounted with Mowiol (Calbiochem, La Jolla, CA, USA) and image acquisition was performed with a Leica TCS SP2 Laser Scanning system (Leica, Mannheim, Germany) attached to a DM IRE-2 inverted microscope with a 40× oil objective (numeric aperture 1.25). The images were processed with Leica LCS software and all the images were assembled using Adobe Photoshop CS2 software.

### Quantitative real-time RT-PCR

Total RNA of the cells was isolated using TRI Reagent RT (Molecular Research Centre, Inc., Cincinnati, OH, USA). One microgram of total RNA was reverse transcribed using a High Capacity RNA-to-cDNA Kit (Applied Biosystems, Foster City, CA, USA) followed by PCR amplification using a Maxima SYBR Green qPCR Master Mix (Fermentas GMBH, St. Leon-Rot, Germany) and a LightCycler II instrument (Roche Diagnostics, Mannheim, Germany). The data were analyzed using Light Cycler software, version 3.5 (Roche Diagnostics, Mannheim, Germany). The expression level of AZIN2 was normalized against either human glyceraldehyde-3-phosphate dehydrogenase (GAPDH) or rat β-2-microglobulin (β2 m). The following primers were used: human AZIN2 (forward, 5′-AGGGGCCAAAGTGAGATTTG-3′; reverse, 5′-CTTGGCAATGATGCTGACTG-3′); rat AZIN2 (forward, 5′-GGGAGCCAAAGTGAGATTTG-3′; reverse, 5′-TGAAGGCTGACGTCACATAG-3′); human GAPDH (forward, 5′-GGTGAAGGTCGGAGTCAAC-3′; reverse, 5′-CAAATGAGCCCCAGCCTTC-3′); rat β2 m (forward, 5′-ATGGCTCGCTCGGTGACC; reverse, 5′-GTGAATTCAGTGTGAGCCAG-3′). The data are presented as mean ± SD of triplicate samples.

### Serotonin and β-hex release assay

LAD2 and *in vitro* generated human MCs were depleted of polyamines by treatment with 2 mM DFMO, a specific inhibitor of ODC, for 90 min to 4 days in IMDM-based medium containing 1% (v/v) dialyzed bovine serum. Cells were sensitized overnight with 1 µg/ml of human myeloma IgE. Reconstitution of polyamines was accomplished by adding 0.5 mM putrescine to the cells 90 min prior to their activation. Degranulation of the sensitized MCs was induced using anti-IgE for 0–30 min at 37°C, after which the conditioned medium was collected and analyzed for serotonin and β-hex. Serotonin was quantitated with commercial ELISA (Labor Diagnostika Nord, Nordhorn, Germany) and β-hex was analyzed using a colorimetric assay as previously described [26]. Briefly, 60 µl of supernatant was added in duplicates into a 96-well plate and mixed with an equal volume of substrate solution (7.5 mM p-nitrophenyl-N-acetyl-β-D-glucosaminide dissolved in 80 mM citric acid, pH 4.5). The plate was thereafter incubated with a gentle agitation for 60 min at 37°C. The reaction was then stopped by addition of 120 µl of glycine (0.2 M, pH 10.7) to each well, and the optical density at 405 nm and 490 nm was determined using a plate reader (Wallac Victor3, Perkin-Elmer, Turku, Finland).

## Results

### Characterization of AZIN2 antisera

**Figure 1:**
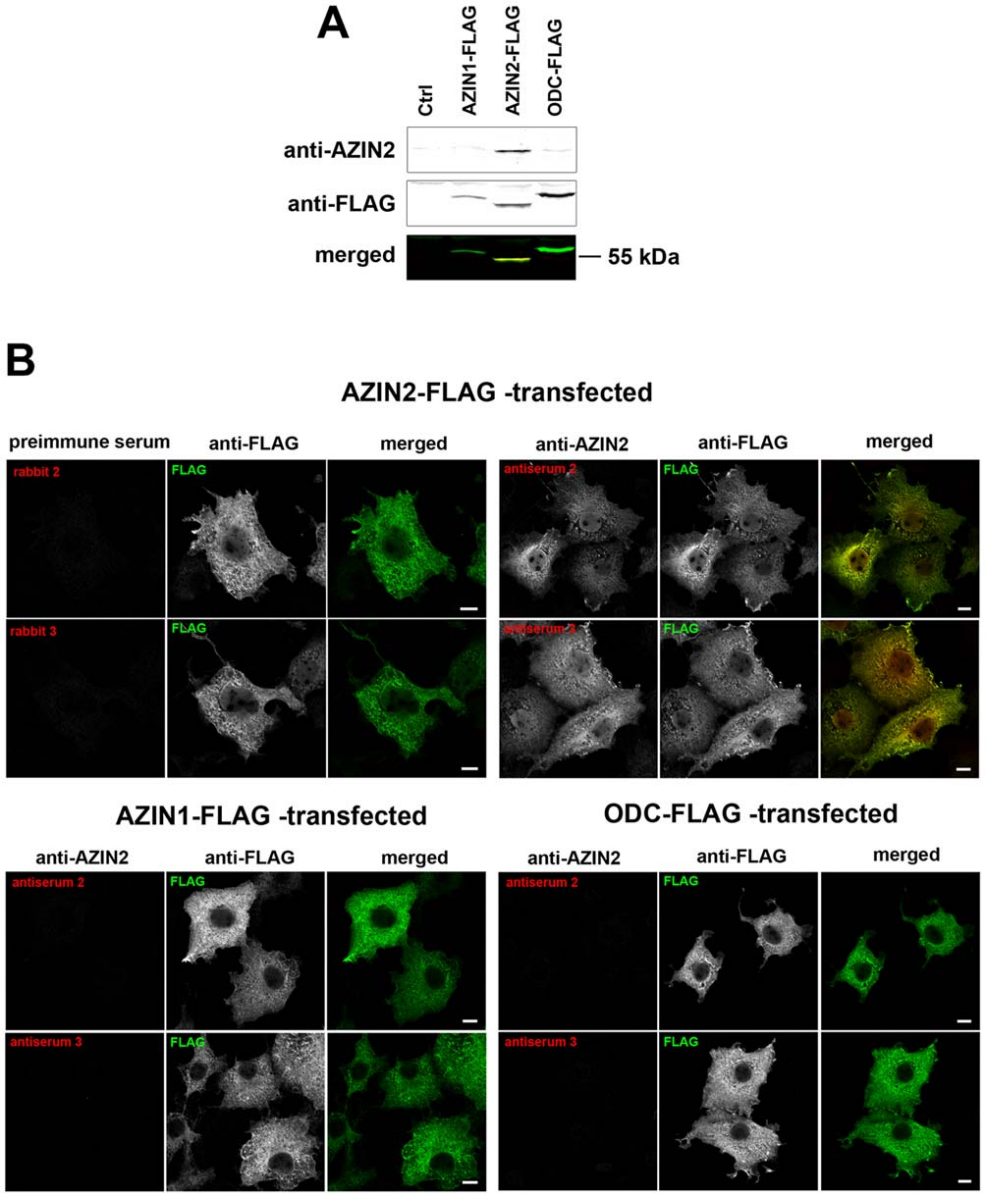
Specificity testing of AZIN2 antisera. (A) Immunoblot from COS-7 cells transfected with plasmids carrying FLAG-tagged AZIN1, AZIN2, or ODC gene or an empty FLAG-vector (Ctrl). An Odyssey Infrared Imager was used for simultaneous two-color detection of rabbit anti-AZIN2 (antiserum 3) and mouse anti-FLAG antibodies. The lowest panel represents merged blots, yellow indicating co-localization of anti-AZIN2 and anti-FLAG. (B) Confocal laser scanning microscopy of COS-7 cells expressing one of the following genes: AZIN2-FLAG, AZIN1-FLAG, or ODC-FLAG. The cells were stained with either AZIN2 antiserum 2 or 3 together with mouse anti-FLAG, followed by donkey anti-rabbit Alexa Fluor-555 and donkey anti-mouse Alexa Fluor-488. AZIN2-FLAG-transfected cells were also stained with preimmune sera of the corresponding rabbits. In the merged images, yellow indicates the co-localization. Bars represent 10 µm.

Antisera 2 and 3 raised against AZIN2 were evaluated for their specificity to AZIN2 to exclude cross-reactivity with the homologous AZIN1 or with ODC. We transfected COS-7 cells with AZIN2, AZIN1, or ODC tagged at the N-terminus with three FLAG epitopes. Cell lysates were separated on SDS-PAGE and immunoblotted with each of the two rabbit antisera together with mouse anti-FLAG antibody. Simultaneous detection of two antibodies was performed using an Odyssey Infrared Imager. Antiserum 3 detected the same ∼55 kDa band in AZIN2-FLAG transfectants as in anti-FLAG without a backround (Figure 1A). No cross-reactivity was observed with either ODC-FLAG or AZIN1-FLAG, indicating specificity of the antiserum to AZIN2. However, antiserum 2 failed to recognize any proteins in immunoblotting, and was therefore retracted from this application (data not shown). To further validate the antisera, COS-7 cells were grown on coverslips, transfected with FLAG-tagged AZIN2, AZIN1, or ODC for 24 h, and stained with mouse anti-FLAG antibody together with either antiserum 2 or 3, and the corresponding preimmune sera. The primary antibodies were detected with fluorescent-labeled, species-selective secondary antibodies, and visualized by confocal microscopy. Both antiserum 2 and antiserum 3 stained COS-7 cells successfully, and the signal co-localized with FLAG in AZIN2-FLAG transfected cells (Figure 1B), whereas no reactivity was seen in AZIN1-FLAG or ODC-FLAG transfectants. Negative control stainings with rabbit preimmune sera showed no positive signal (Figure 1B).

### Expression of AZIN2 in cutaneous mastocytosis

**Figure 2:**
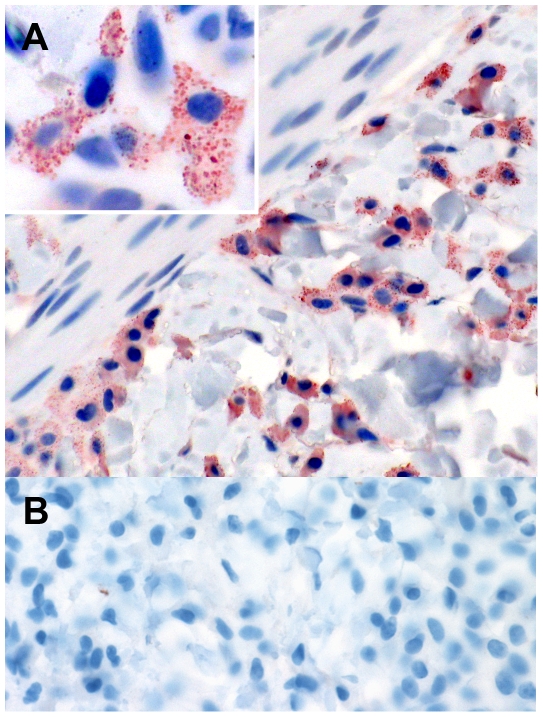
Immunohistochemical identification of AZIN2 protein in human cutaneous mastocytosis. Human skin tissue sections stained histochemically with either antiserum 3 to AZIN2 (A) or rabbit preimmune serum (B). Original magnifications: ×400 (A–B) and ×1000 (inset). Note the cytoplasmic granular-like staining pattern of AZIN2 in the inset.

Sections of human skin samples containing neoplastic infiltrates of MCs were immunohistochemically stained with the two rabbit antibodies to AZIN2. Positive staining for AZIN2 was selectively detected in the MCs of the affected skin areas, and also in scattered normal skin MCs (Figure 2A). AZIN2 localized in the cytoplasm with punctated, granular distribution (Figure 2A, inset). Both rabbit antiserum 2 and 3 showed an identical staining pattern. Preimmune sera from the corresponding rabbits did not stain positively (Figure 2B).

### Granular expression of AZIN2 in MCs

**Figure 3:**
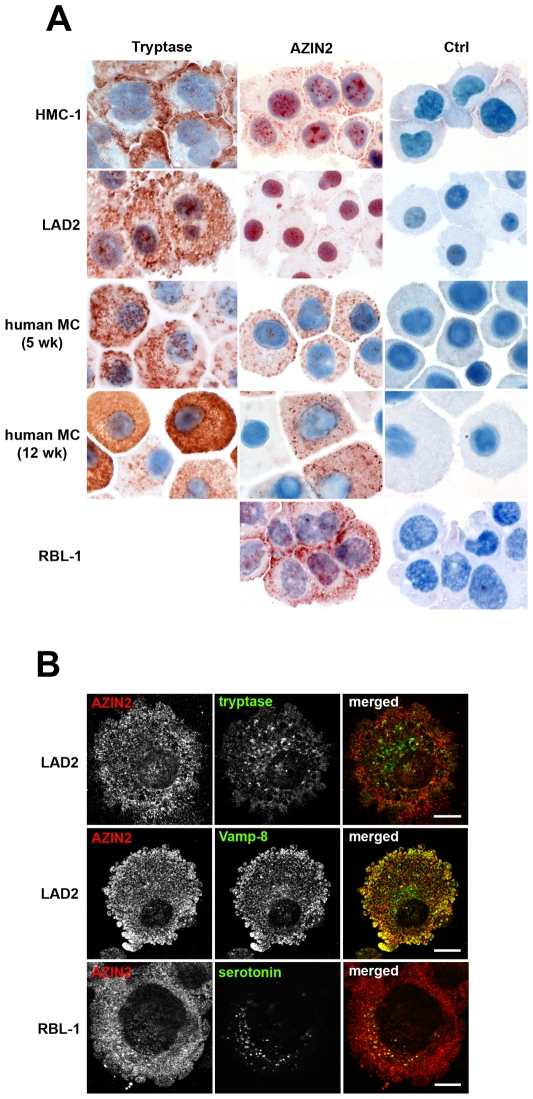
Granular expression of AZIN2 in MC lines and primary human MCs. (A) The MCs were immunostained with either anti-tryptase, AZIN2 antiserum 3 (HMC-1, LAD2, and RBL-1), antiserum 2 (primary human MCs) or the corresponding rabbit preimmune sera (Ctrl). Note the high variation of AZIN2 expression between individual human MCs cultured for 12 weeks. Original magnification for all panels ×1000. (B) Confocal laser scanning microscopy for AZIN2 with MC granule markers tryptase and Vamp-8 in LAD2 cells, and serotonin in RBL-1 cells. Double-immunofluorescence staining was performed using rabbit antiserum 2 together with monoclonal antibody to tryptase, mouse anti-Vamp-8 or monoclonal antibody to serotonin, followed by Alexa Fluor-555 conjugated donkey anti-rabbit and Alexa Fluor-488 conjugated donkey anti-mouse IgGs. The right panel represents merged images; yellow indicates the co-localization. Bars represent 10 µm.

We next investigated the possible expression of AZIN2 in human and murine MC lines. Immunocytochemical stainings were performed on cytocentrifuged rat (RBL-1) and human (HMC-1 and LAD2) MCs using the two rabbit antisera against AZIN2 and the corresponding preimmune sera. To identify MC granules, we also stained the MCs with monoclonal antibody to tryptase. In all MC lines tested, AZIN2 and tryptase localized in the cytoplasm with a granular pattern, revealing their presence in the secretory granules (Figure 3A). AZIN2 was typically located in the vicinity of the plasma membrane. The RBL-1 cells showed the highest and LAD2 cells the lowest staining intensity for AZIN2. Identical staining patterns were obtained with both antisera. In addition, some nuclear AZIN2 was seen in both murine and human MCs. All control stainings with the preimmune sera were negative (Figure 3A). The expression of AZIN2 mRNA in these MC lines was also verified by quantitative real-time PCR (data not shown). Staining of HMC-1 and LAD2 cells for tryptase revealed greater amounts of tryptase-positive granules than of AZIN2-positive granules (Figure 3A). Since we used a human tryptase antibody that did not cross-react with murine tryptase, we were unable to perform staining for tryptase in RBL-1 cells.

The MC lines used in the study originated from neoplasias. We repeated the immunocytochemical stainings on benign, primary human MCs that had been differentiated *in vitro* for 5 and 12 weeks from adult peripheral blood CD34+ progenitor cells. Immature 5-week-old primary human MCs expressed AZIN2 uniformly throughout the cytoplasm in a granular pattern (Figure 3A), similar to that seen in the MC lines. A similar granular distribution of AZIN2 was also seen in the 12-week-old mature human MCs, but with considerable variation in the level of AZIN2 expression among the individual cells. It was also notable that the AZIN2-positive granules in the primary MCs were more evenly distributed throughout the cytoplasm, and not clustered in the periphery as in the MC lines. The primary human MCs also contained more of tryptase-positive granules than AZIN2-positive granules (Figure 3A). Furthermore, variable amounts of tryptase were observed in the mature human MCs, similar to what was seen in AZIN2, suggesting that extended differentiation *in vitro* induces heterogeneity in the MCs.

We performed double immunofluorescence stainings to characterize further the AZIN2-containing granules. Simultaneous staining of LAD2 cells with antibodies to AZIN2 and tryptase did not reveal any co-distribution, indicating that tryptase and AZIN2 localize in distinct granules. Co-staining of AZIN2 and serotonin showed that all serotonin-containing granules were also positive for AZIN2 in RBL-1 cells. In the LAD2 cells, AZIN2 expression overlapped partially with Vamp-8, especially in the peripheral cytoplasm, close to the plasma membrane (Figure 3B). Vamp-8 has been shown to localize specifically in serotonin-containing MC granules [27]. This finding, together with the present data, shows that AZIN2 is selectively expressed in the Vamp-8 positive, serotonin-containing subset of MC granules, suggesting a granule subset-specific function.

### AZIN2 expression is coupled to activation of MCs

**Figure 4:**
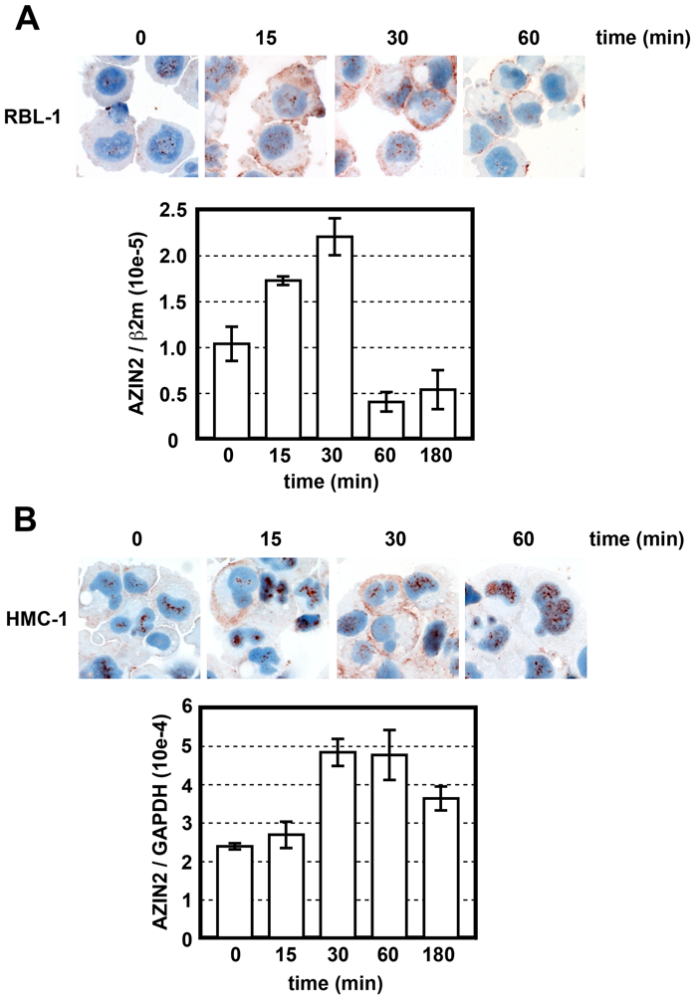
Upregulation and redistribution of AZIN2 in MCs during activation. RBL-1 (A) and HMC-1 (B) cells were activated with PMA and A23187 for the times indicated. After activation, the cells were either immunostained for AZIN2 (antiserum 2) or harvested for quantitative real-time PCR analysis of AZIN2. Relative mRNA levels are expressed as the ratio of AZIN2 mRNA level to that of either rat β-2-microglobulin (β2 m) or human glyceraldehyde-3-phosphate dehydrogenase (GAPDH). Original magnification of all immunostainings: ×400.

Next we examined whether the expression of AZIN2 is coupled to the activation of MCs. The expression of AZIN2 mRNA was quantified by real-time PCR, and the distribution of AZIN2 positive granules was studied by immuocytochemistry in RBL-1 and HMC-1 cells, after the cells had been activated with PMA and the calcium ionophore A23187. After 15 min of activation, the mRNA of AZIN2 was elevated in both cell lines; after 30 min the mRNA levels had more than doubled as compared to untreated control cells (Figure 4A–B). Thereafter, AZIN2 mRNA expression declined, and after 180 min the RBL-1 cells expressed even less AZIN2 than the untreated cells (Figure 4A). A similar decrease in the level of AZIN2 mRNA was also seen in HMC-1 cells after 180 min of activation (Figure 4B). This rapid fluctuation of AZIN2 mRNA suggests a functional relation of AZIN2 transcription with MC activation.

Immunocytochemistry of activated RBL-1 and HMC-1 cells revealed changes in AZIN2 staining intensity and also in the distribution of AZIN2-positive granules (Figure 4A–B). In untreated RBL-1 and HMC-1 cells, AZIN2 was detected in sparse, dispersed cytoplasmic granules and also in the nucleus. After 15 min of activation, the staining intensity for AZIN2 was increased in both the cytoplasm and the nucleus. AZIN2-positive granules relocated to the peripheral areas of the cells. The staining intensity for AZIN2 peaked after 30 min of activation, and translocation of AZIN2-positive granules to the vicinity of the plasma membrane became more prominent. After 60 min of activation, AZIN2 was seen only on the cell membrane of a few RBL-1 cells (Figure 4A), whereas the HMC-1 cells expressed AZIN2 solely in the nucleus (Figure 4B). These activation-induced rapid changes in the expression level and intracellular distribution suggest that AZIN2 plays a role in the exocytosis of MC granules.

### Polyamine depletion delays serotonin release from MCs

**Figure 5:**
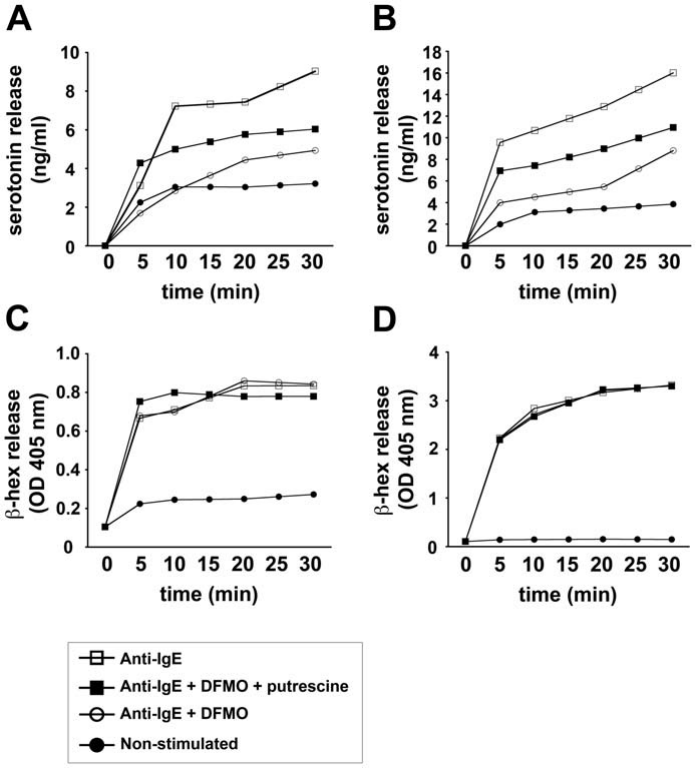
Polyamine depletion inhibits serotonin release from MCs. LAD2 (A, C) and primary human MCs (B, D) were cultured in polyamine-free medium and sensitized overnight with human myeloma IgE. Before activation, the cells were and treated with 2 mM DFMO as described in Materials and Methods, and subsequently activated with anti-IgE for the indicated times; and the conditioned medium was analyzed for serotonin (A–B) and β-hex (C–D) release. Symbols represent treatment of MCs: anti-IgE only (□), anti-IgE + 2 mM DFMO + 0.5 mM putrescine (▪), anti-IgE + 2 mM DFMO (○), non-stimulated (•).

Given that AZIN2 is an activator of ODC, the rate-limiting enzyme of polyamine biosynthesis, we next investigated the impact of polyamines on MC degranulation. Primary human MCs and LAD2 cells were cultivated in polyamine-free medium and treated with DFMO to block ODC activity. Treatment with DFMO inhibited the IgE-triggered release of serotonin from both primary human MCs and LAD2 cells (Figure 5A–B). Furthermore, this inhibitory effect of DFMO was reversed by the addition of putrescine to the cells. In contrast, treatment with DFMO with or without putrescine, had no impact on β-hex (Figure 5C–D) or histamine release (data not shown). We extended the duration of DFMO treatment from 90 min to 4 days prior to activation of the cells. The inhibitory effect on induced serotonin release persisted, whereas the release of β-hex and histamine remained unchanged, suggesting that the basal level of polyamines is low in resting MCs. All in all, these results indicate that polyamines selectively regulate the release of serotonin from MCs without affecting the release of other granular MC mediators. Our serotonin release assay also showed that the content of serotonin was higher in primary human MCs than in LAD2 cells (Figure 5A–B).

## Discussion

AZINs function as inducers of ODC activity in the biosynthesis and uptake of polyamines. Of the AZINs, AZIN1 is ubiquitously expressed and found in all mammalian cells. The other isoform, AZIN2, which we originally discovered (then called ODCp), has a selective distribution, and was previously reported to be present only in the brain and testis [19]. In the present study, we report a robust expression of AZIN2 in normal and neoplastic MCs.

In our original paper on human AZIN2 [19], we reported the possibility of at least eight different splicing variants for this protein. The biological significance of the alternative splicing and the amount of *in vivo* translated splicing variants of AZIN2 remains to be established. The likely existence of splicing variants of AZIN2 may nevertheless have an impact on its reactivity with antibodies. We used two rabbit antisera raised against peptides representing the most unique part of AZIN2. The peptide used as immunogen for antiserum 2 was derived from a sequence that spans an intron-exon boundary known to involve multiple splicing, beginning from exon 1 and ending in exon 3, but excluding exon 2, which is not included in the splicing variants 1–7 [19]. The second peptide used to produce antiserum 3 was derived from exon 1, present in all putative splicing variants of AZIN2. As seen in Figures 3 and 4, the staining intensity of AZIN2 varied slightly between the two antisera, antiserum 3 producing stronger stainings than antiserum 2. This may be due to alternative splicing of AZIN2. In spite of the variations in the staining intensities, both antiserum 2 and 3 yielded highly similar results regarding the staining patterns of MC granules, while only antiserum 3 was functional in immunoblotting. Due to the high degree of structural homology between ODC and AZINs, earlier studies with antisera raised against the whole ODC protein may need to be reinterpreted, due to the potential cross-reactivity with AZIN1 and AZIN2.

Immunocytochemical stainings for AZIN2 with antisera 2 and 3 revealed a granular distribution mainly in the cytoplasm of primary human MCs and the MC lines. Immunohistochemistry of normal and neoplastic tissue MCs showed a similar granular pattern. Immature human primary MCs obtained after 5 weeks of culture displayed a homogenous expression of AZIN2-positive granules, whereas 12-week-old mature MCs varied considerably in the intracellular expression of AZIN2-positive granules. This suggests that the terminal differentiation of MCs *in vitro* leads to the establishment of a subpopulation of MCs with variable expression of AZIN2. In addition to the cytoplasmic granules, there were variable amounts of AZIN2 in the nuclei of MCs. Especially antiserum 3 detected frequently nuclear AZIN2 in all the MC lines used regardless of the staining method. Since ODC, AZs, and polyamines have been shown to localize also in the nuclei of different mammalian cells [28]–[30], the nuclear staining for AZIN2 was not entirely unexpected, although its functional relevance remains unclear. ODC has a rapid turnover in normal cells, and AZIN1 is also known for its short half-life [31]. We found a promptly upregulated expression of AZIN2 mRNA upon activation of MCs with PMA and the calcium ionophore A23187. Although the half-life of AZIN2 remains to be determined, our findings suggest a rapid turnover of AZIN2 similar to that of its homologues ODC and AZIN1.

We co-stained the AZIN2-containing MC granules with tryptase, a robust marker of classical secretory granules in MCs, in order to further characterize the granules. The staining revealed a distribution of AZIN2 distinct from that of tryptase. Instead, we observed a co-localization of AZIN2 and the vesicle-associated v-SNARE protein Vamp-8 (also known as endobrevin). Moreover, the granular distribution of serotonin overlapped with that of AZIN2. Our results thus support the existence of distinct subsets of MC granules, demonstrated earlier by Puri and Roche [27]. The authors reported an association between subsets of MC granules and specific SNARE proteins, and found that a majority of the serotonin-containing MC granules were Vamp-8-positive. Furthermore, the authors found that serotonin and histamine were present in two distinct granule subtypes, and described that the secretion of serotonin was impaired, but not that of histamine, in MCs of Vamp-8-deficient mice. Differential release of MC mediators further supports the existence of separate granule populations. Already in 1982, Theoharides et al. [32] found evidence for differential release of MC mediators from the secretory granules. The authors showed that amitriptylin blocks the exocytotic release of histamine from activated MCs without affecting serotonin release. Here, we show that in the absence of external polyamines, the treatment of MCs with DFMO, that specifically blocks the activity of ODC, selectively impairs the release of serotonin without affecting the exocytosis of β-hex or histamine. The finding is in agreement with previous reports indicating differential release of MC mediators. We also demonstrate that addition of external putrescine restores the secretion of serotonin, indicating that polyamines are key mediators of serotonin release from MCs.

The expression of AZIN2 in only a few tissue and cell types suggests a specific regulatory role in polyamine homeostasis. In MCs, the induction of AZIN2 expression together with the granule subtype-specific distribution suggests that AZIN2 is likely to mediate ODC activation *in situ* in serotonin-containing granules during MC activation. Since we also provide evidence indicating that secretion of serotonin from MCs following IgE-mediated stimulation is polyamine-dependent, it can be speculated that AZIN2 specifically regulates this process. To further study this potential role of AZIN2, we attempted to knock down AZIN2 by siRNA-mediated RNA interference in MCs. This approach was, however, hampered by the fact that mature MCs turned out to be very difficult to transfect by conventional means. Thus further studies are needed to directly define the role of AZIN2 in relation to serotonin secretion from MCs.

The exact molecular mechanisms by which polyamines selectively regulate the secretion of serotonin from activated MCs remain to be elucidated. However, there are a couple of potential mechanisms that could be hypothesized. Polyamines regulate the gating of various ion channels including calcium channels, enhancing Ca^2+^ influx in various types of cells [33]–[37]. Activation of MCs with ensuing granule exocytosis is known to depend strongly on Ca^2+^ signaling [5]. Therefore, AZIN2 could participate in local activation of ODC sequestered to AZ, and provoke the production of polyamines *in situ*, which would consequently enhance Ca^2+^ mobilization. On the other hand, granule exocytosis from activated MCs is regulated by small GTP-binding proteins of the Rho family. Price et al. [38] reported that introduction of constitutively active mutants of RhoA and Rac to permeabilized MCs induced enhanced exocytosis upon triggering, whereas inactivation of RhoA by pretreatment with the *Clostridium botulinum* C3 transferase abolished the effect. Although the RhoA family proteins are the major modulators of actin cytoskeleton dynamics, their impact on MC exocytosis is not directly linked to the regulatory effects on actin polymerization [38]. We have recently reported that RhoA is activated *in vivo* by transglutaminase-2-mediated polyamination in an ODC-dependent fashion [39]. Given this, it is appealing to speculate that AZIN2-induced local activation of ODC and polyamine biosynthesis may lead to *in situ* polyamination and activation of RhoA, which subsequently governs the intracellular transport of MC granules.
